# Clinical and genetic characterization of Lenz-Majewski syndrome with a PTDSS1 variant: a case report and literature review

**DOI:** 10.3389/fped.2025.1617541

**Published:** 2025-08-05

**Authors:** Yahua Zhang, You Wu, Lulu Yan, Yuxin Zhang, Haibo Li, Yan He

**Affiliations:** ^1^ Department of Infectious Diseases, Women and Children’s Hospital of Ningbo University, Ningbo, Zhejiang, China; ^2^ Department of Public Health, Women and Children’s Hospital of Ningbo University, Ningbo, Zhejiang, China; ^3^ The Central Laboratory of Birth Defects Prevention and Control, Women and Children’s Hospital of Ningbo University, Ningbo, Zhejiang, China; ^4^ Ningbo Key Laboratory for the Prevention and Treatment of Embryogenic Diseases, Women and Children’s Hospital of Ningbo University, Ningbo, Zhejiang, China; ^5^ Department of Neurology, Women and Children’s Hospital of Ningbo University, Ningbo, Zhejiang, China

**Keywords:** PTDSS1 gene, whole exome sequencing, Lenz-Majewski syndrome, clinical manifestations, genetic analysis

## Abstract

**Introduction:**

Lenz-Majewski syndrome (LMS) is an ultra-rare congenital disorder with progressive skeletal dysplasia, cutis laxa, and intellectual disability, typically caused by pathogenic variants in the PTDSS1 gene.

**Methods:**

Our patient with multiple malformations and developmental delay who was treated at the Women and Children's Hospital of Ningbo University in September 2023 was selected as the research subject. Whole exome sequencing (WES) technology was used to test the child, and Sanger sequencing verification and pathogenicity analysis were carried out for the suspected variations.

**Results:**

We report the first molecularly confirmed case of LMS in a Chinese patient, a male infant presenting with classic features such as craniofacial dysmorphism, hyperostosis, loose skin, syndactyly, and short stature, together with mild global developmental delay and thyroid dysfunction. Whole exome sequencing (WES) identified a heterozygous c.806C > T (p. Pro269Leu) variant in the PTDSS1 gene, which was validated by Sanger sequencing and functionally assessed for pathogenicity. We further reviewed 12 previously reported cases with PTDSS1 variants and compared phenotypes, highlighting both shared and unique features.

**Discussion:**

This case expands the ethnic and phenotypic spectrum of LMS and reinforces the association between the c.806C > T (p. Pro269Leu) variant and LMS. Early genetic testing facilitates recognition of atypical presentations and enables timely diagnosis and management.

## 1 Introduction

Lenz-Majewski syndrome (LMS, MIM: #151050), or Lenz-Majewski hyperostotic dwarfism, is a rare syndrome of intellectual disability, sclerotic bone dysplasia, facial dysmorphism, brachydactyly, syndactyly, and cutis laxa (1). In 1969, the syndrome was first described by Braham (2). And then delineated as a distinct form of hyperostotic dwarfism with a progeroid appearance by Lenz and Majewski in 1974 (3). LMS has an autosomal dominant mode of inheritance (4). The condition is caused by variants of the PTDSS1 gene that encodes phosphatidylserine synthase 1 (PSS1), an enzyme involved in phosphatidylserine biosynthesis. Pathogenic variations in PTDSS1 lead to enzyme dysregulation, resulting in excessive phosphatidylserine production and loss of normal end-product feedback inhibition. This leads to a gain-of-function (GOF) effect and aberrant fibroblast metabolism (5). Twelve patients with PTDSS1 variants have been described in the literature so far (1, 5, 6–12). Herein, we report the first molecularly confirmed case of LMS in a Chinese patient, a male infant who presented with generalized cutis laxa, distinctive craniofacial and digital malformations, and global developmental delay. Whole exome sequencing (WES) was employed to determine the underlying genetic etiology and to establish a basis for genetic counseling for this family. In addition to the common features, the patient also presented with mild intellectual disability and an unusual combination of skeletal and cutaneous manifestations. These manifestations further expand the established phenotypic spectrum of LMS. The case adds valuable information to the limited existing literature on LMS and underscore the need for greater awareness and genetic screening among underrepresented populations.

## 2 Case presentation

An 8-month-old male patient was admitted to the Women and Children's Hospital of Ningbo University in September 2023 due to deviation of the left oral commissure when crying. He was the first child of the mother's second pregnancy (gravida 2, para 1) and was full-term, with spontaneous vaginal delivery. His birth weight was 2,700 g, length was 46 cm (below the 5th percentile), and head circumference was 31.5 cm (within the normal range), consistent with intrauterine growth restriction (IUGR). There was no history of perinatal asphyxia, cyanosis, and need for resuscitation. A potential IUGR was diagnosed during pregnancy in his mother. Apgar scores were 10 at 1 and 5 min of age, and breastfeeding was initiated shortly after birth.

**Figure 1:**
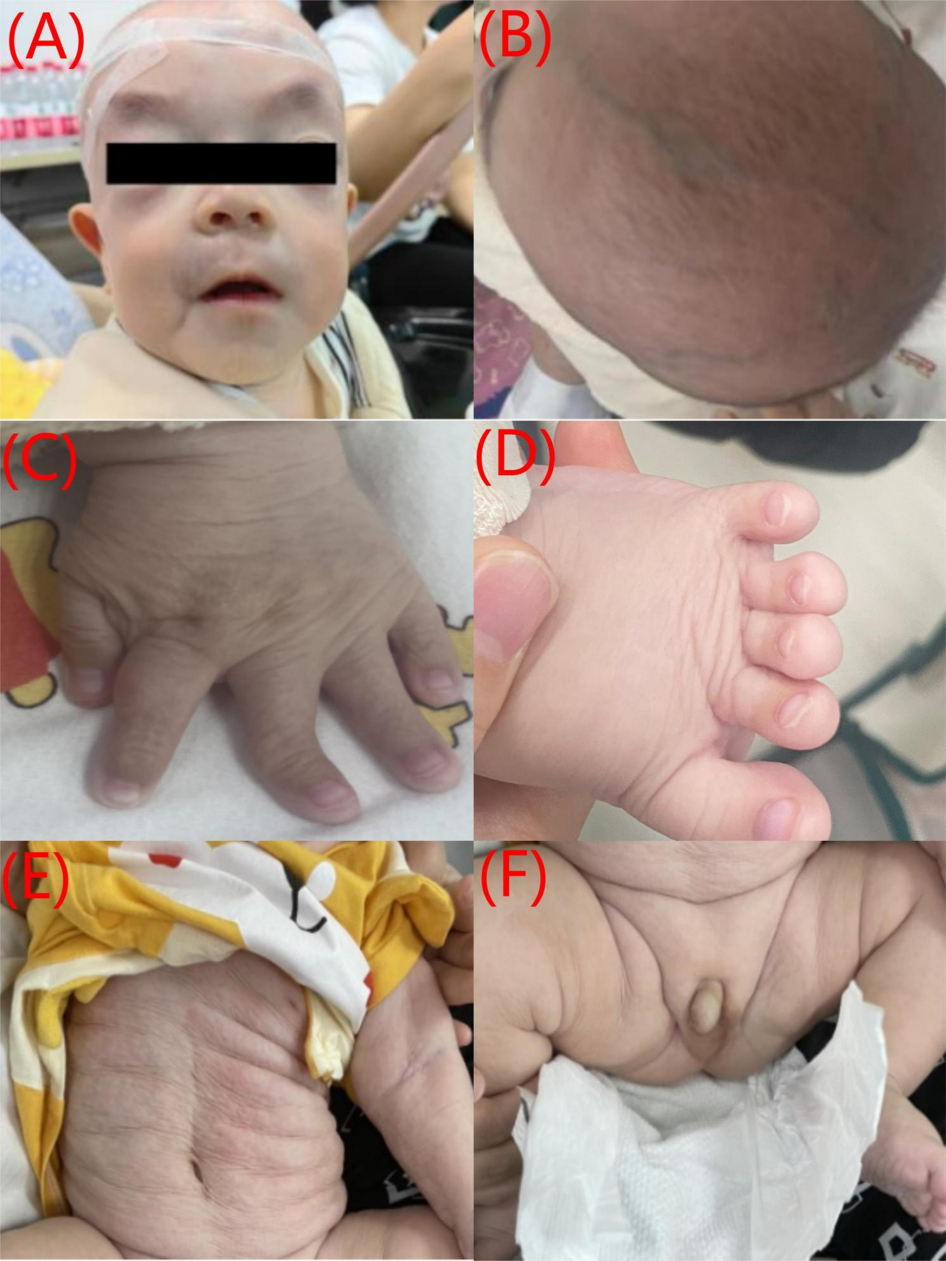
A collage of six images showing different parts of a baby's body. **(A)** Front view of a baby's face with a hairnet and eyes blacked out. **(B)** Top view of the baby's head showing hair growth. **(C)** Close-up of the baby's hand, showing the fingers. **(D)** Close-up of the baby's foot, showing the toes. **(E)** Side view of the baby's abdomen with visible skin folds. **(F)** Baby's abdomen with visible folds and the diaper partially open., and father. Each graph displays colored peaks representing nucleotides C, T, A, and G. Red arrows highlight differences in sequences.

The patient presented with multiple congenital malformations at birth. The dysmorphic features of the face included macrocephaly, upturned nostrils, long and smooth philtrum, and thin lips (Figure 1A). The other features included hypertelorism, downward-slanting palpebral fissures, a flat nasal bridge (Figure 1B), a large anterior fontanelle, and prominent scalp veins (Figure 1C). Cryptorchidism was also noticed (Figure 1D). His fingers were rigid and had varying levels of deformity such as deviation of the left middle finger, short fifth finger, and syndactyly (Figure 1E). The skin was wrinkled and atrophic over the entire body (Figure 1F). Developmentally, the child had generalized delay. He could lift his head while prone but could not roll over. He did not have any specific response to auditory stimuli such as a ringing bell, could not fixate on the human face, and did not follow moving objects. The Gesell Developmental Scale tests showed the following development quotients and age: adaptive development quotient 16 (1.3 months), gross motor development quotient 43 (2.5 months), fine motor quotient 53 (3 months), language quotient 71 (4 months), and personal-social quotient 36 (2 months). Ophthalmologic and auditory exams were unremarkable.

**Figure 2:**
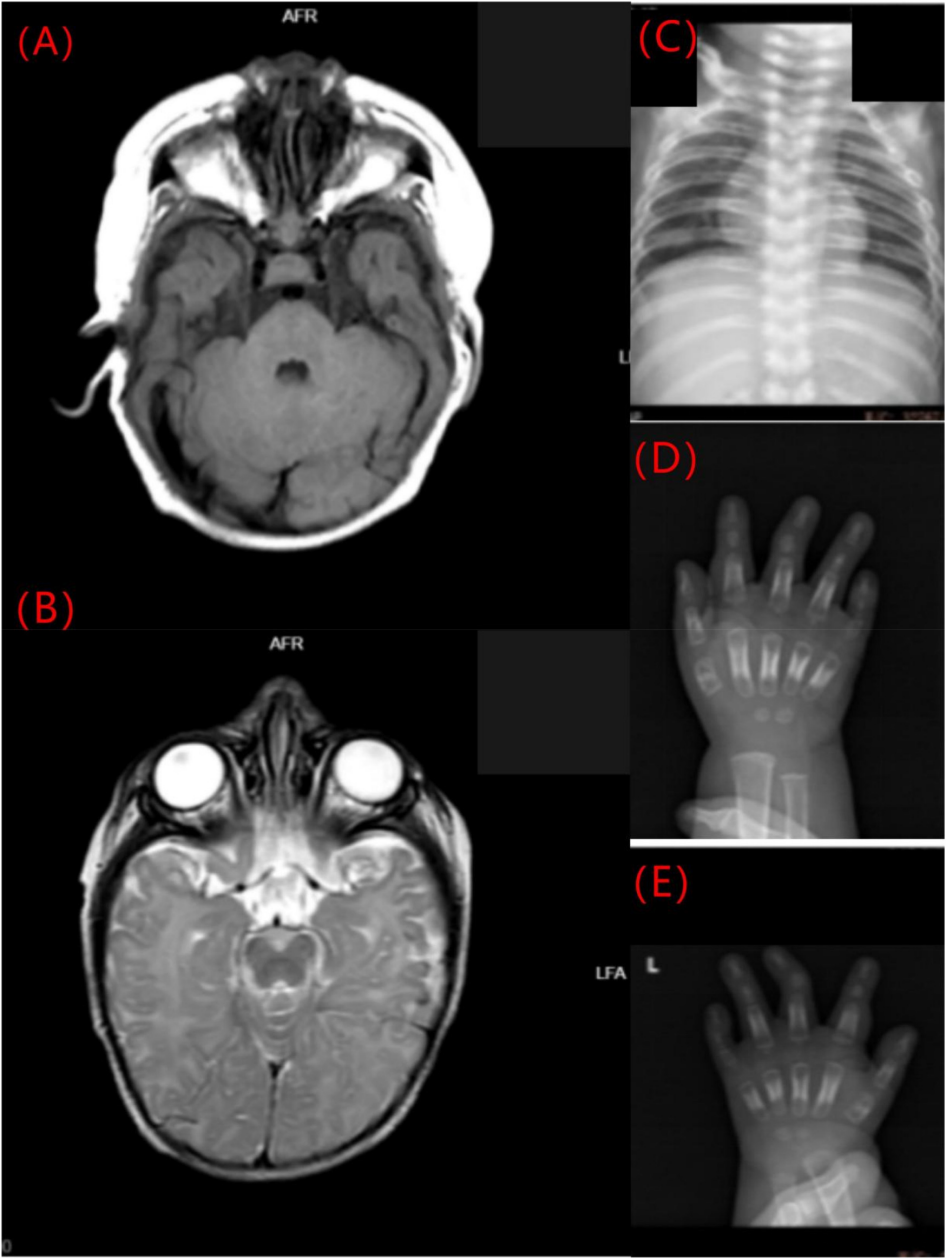
Imaging findings of the LMS patient. **(A)** Mild hemosiderin deposition in the left cerebellar hemisphere (axial MRI). **(B)** Focal cerebral atrophy with surface hemosiderin deposits in the left frontal lobe (axial MRI). **(C)** Thickened chest ribs and cortical bone (anteroposterior radiograph). **(D)** Deformed right-hand phalanges with cortical thickening and irregular contours (anteroposterior radiograph). **(E)** Abnormal morphology and cortical thickening of the middle phalanges of the left hand (anteroposterior radiograph).

**Figure 3:**
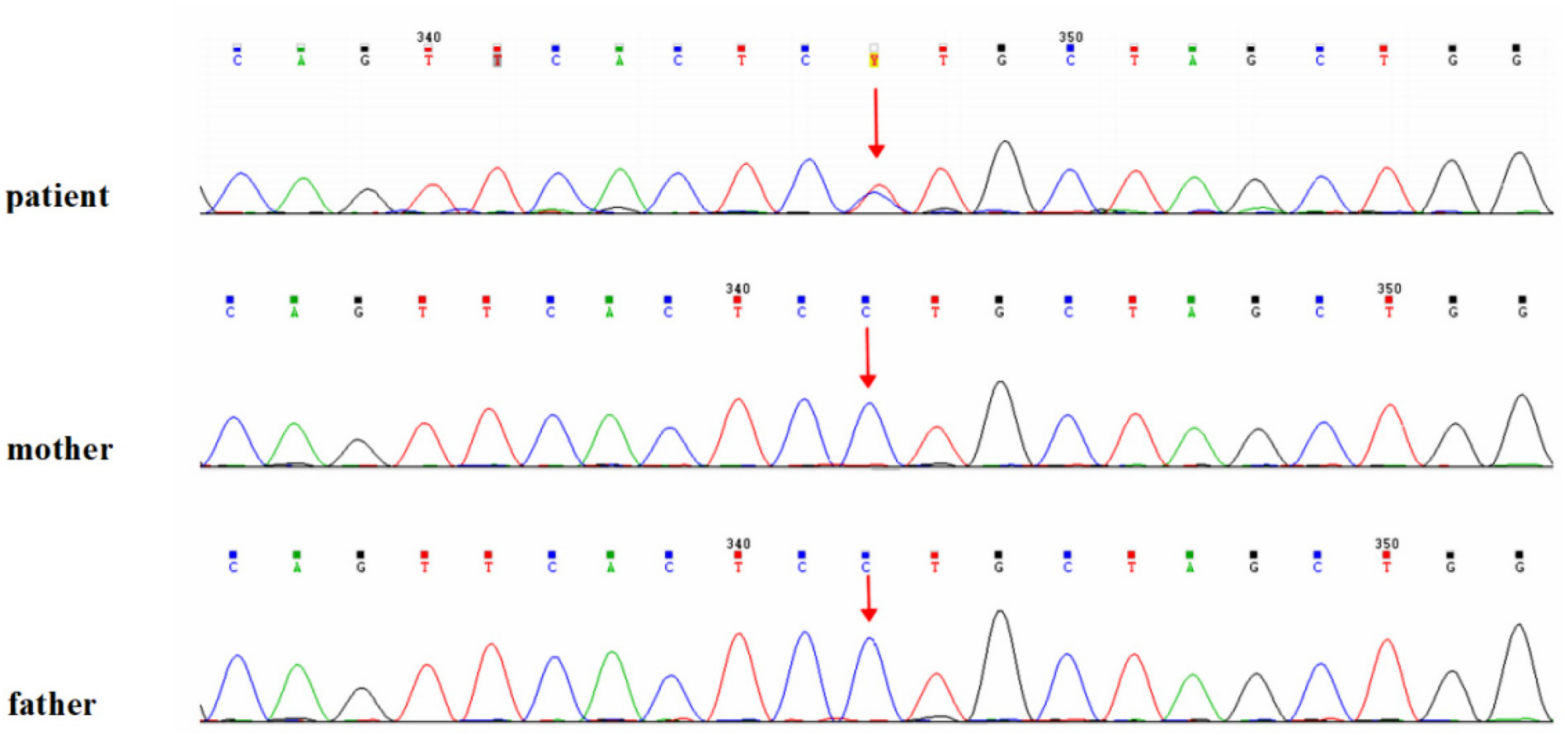
Genetic analysis of the patient. Identification of the heterozygous c.806C > T (p.Pro269Leu) variant in the PTDSS1 gene by whole-exome sequencing, confirmed by Sanger sequencing.

**Figure 4:**
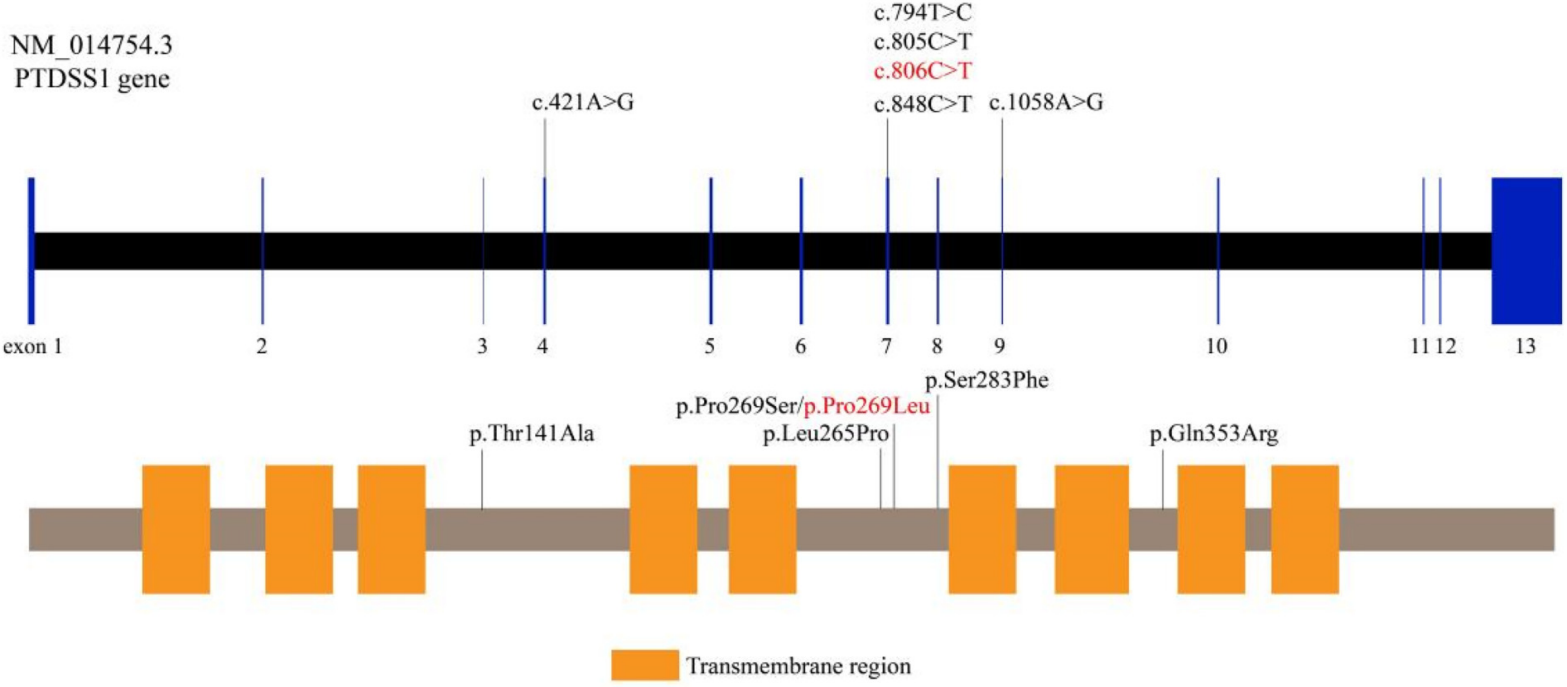
Pathogenicity assessment of the PTDSS1 c.806C > T (p.Pro269Leu) variant. The variant is absent from population databases, confirmed as *de novo* by parental Sanger sequencing, and predicted to be damaging by multiple in silico tools. The patient's phenotype is consistent with the clinical features of LMS.

Cranial MRI demonstrated mild deposition of hemosiderin in the left cerebellar hemisphere (Figure 2A) and focal cerebral atrophy with surface hemosiderin deposits in the left frontal lobe (Figure 2B). Radiographs demonstrated thickening of the chest ribs and cortical bone (Figure 2C). The phalanges of the right hand were deformed, exhibiting cortical thickening and irregular contours from the second to fifth phalanges (Figure 2D). The left-hand middle phalanges of the second to fifth fingers also displayed abnormal morphology, including curvature of the third phalanx, and cortical thickening of metacarpal and phalangeal bones (Figure 2E). Echocardiography showed a 1.5 mm patent foramen ovale. Comprehensive thyroid function tests showed a slightly elevated total thyroxine (TT4) of 162.56 nmol/L (reference range: 78.4–157.4 nmol/L) and free thyroxine (FT4) of 19.66 pmol/L (11.2–18.1 pmol/L). The total triiodothyronine (TT3) was mildly low at 1.22 nmol/L (1.34–2.73 nmol/L), while free triiodothyronine (FT3) was within normal limits at 4.04 pmol/L (3.67–10.43 pmol/L). Thyroid-stimulating hormone (TSH) was 2.98 mIU/L (0.2–7.0 mIU/L), still within the normal reference range. Liver, kidney, and cardiac function tests were normal. The parents were non-consanguineous, healthy, and denied any family history of genetic disorders. WES and Sanger sequencing identified that the patient carried a heterozygous variant c.806C > T (p. Pro269Leu) in the PTDSS1 gene (NM_014754.3) (Figure 3). The variant was not observed in either of the parents, indicating a *de novo* variant. The variant was categorized as pathogenic according to the ACMG guidelines. It is not present in various population databases, including 1,000 Genomes Project, ExAC, gnomAD, and dbSNP (PM2), as well as from the ClinVar database. This supports its rarity and fulfills the ACMG PM2 criterion as moderate evidence of pathogenicity. Sanger sequencing confirmed that the variant was not present in either parent, indicating a *de novo* origin (PS2). Multiple in silico prediction tools support its pathogenicity: REVEL score of 0.82, ClinPred score of 0.9999, and both SIFT,and PolyPhen-2 predicted a damaging effect (PP3). The patient's phenotype is very concordant with the LMS clinical features (PP4) (Figure 4).

## 3 Discussion

LMS is an extremely uncommon genetic disorder first described by Lenz and Majewski in 1974 (13). It is characterized by a constellation of clinical features that include intellectual disability, progressive hyperostosis, cutis laxa, amelogenesis imperfecta, craniofacial abnormalities, and digital malformations. In 2014, Sousa et al. identified GOF variants in the PTDSS1 gene in five sporadic patients with LMS, a breakthrough in its molecular etiology (9). So far, a total of 12 molecularly confirmed cases have been reported in the literature, including five males and seven females (1, 5, 6–12). Herein, we report the first molecularly confirmed Chinese patient with LMS, expanding the known ethnic and geographic distribution of the disorder. Our patient had a heterozygous c.806C > T (p. Pro269Leu) variant in the PTDSS1 gene, which was the identical variant previously identified in a patient of European ancestry by Piard et al. (7). Interestingly, our patient also manifested some novel or less commonly reported features, including mild intellectual disability, absence of both proximal interphalangeal synostosis and choanal atresia, thyroid dysfunction, and mild cerebral atrophy. Moreover, though our patient existed with limited behavioral responses to visual and auditory stimuli, apparently because of developmental delay, formal ophthalmologic and audiologic testing was within normal limits, with no structural or functional defects. This finding suggests that sensory impairment may not be a significant part of LMS, but functional limitations due to neurological involvement cannot be excluded. The results not only expand the LMS phenotype but also underscore the importance of considering this diagnosis in patients with atypical or incomplete presentations, particularly in underrepresented populations such as East Asians.

A systematic review of the literature of all previously documented LMS cases with proven PTDSS1 variants demonstrated a uniform pattern of clinical manifestations. The most frequent features were broad and prominent forehead, delayed closure of the fontanelles, wide interorbital distance (hypertelorism), large and floppy ears, generalized cutis laxa with marked skin atrophy, prominent superficial veins, joint laxity, brachydactyly, progressive skeletal sclerosis, hyperostotic changes of the diaphysis, growth retardation, and intellectual disability. All occurred in 100% of the cases. Proximal interphalangeal synostosis occurred in 92.31% of the patients. Additional features were choanal atresia (75%) and sparse hair (also 75%). Despite differences in clinical presentations, generalized wrinkled and atrophic skin emerged a constant and characteristic finding in all patients (Table 1).

**Table 1:** Clinical phenotypes of previously reported cases and cases in this report.

Clinical features	[1]	[2]	[3]	[4]	[4]	[5]	[6]	[7]	[7]	[7]	[8]	[9]	Present
Prominent forehead	+	+	+	+	+	+	+	+	+	+	+	+	+
Delayed closure of fontanelles	ND	+	+	+	+	+	+	+	+	+	+	+	+
Hypertelorism/Telecanthus	+	+	+	+	+	+	+	+	+	+	+	+	+
Large floppy ears	+	+	+	ND	ND	+	+	+	+	ND	ND	+	+
Choanal atresia	ND	+	ND	ND	ND	+	+	ND	ND	ND	ND	-	ND
Sparse hair	-	-	+	ND	ND	+	+	+	ND	ND	+	+	ND
Loose, atrophic skin/Cutis laxa	+	+	+	+	+	+	+	+	+	+	+	+	+
Prominent cutaneous veins	+	+	+	ND	ND	+	+	+	+	ND	+	+	+
Joint laxity	+	ND	+	+	+	+	+	+	ND	ND	ND	+	+
Brachydactyly	+	+	+	+	+	+	+	+	+	+	+	+	+
Proximal symphalangism	+	+	+	+	+	+	+	-	+	+	+	+	+
Progressive sclerosis	+	+	+	+	+	+	+	+	+	+	+	+	ND
Diaphyseal hyperostosis	+	+	+	+	+	+	+	+	+	+	+	+	+
Growth retardation	+	+	+	ND	ND	+	+	+	+	+	+	+	+
Intellectual disability	+	+	+	ND	ND	+	+	+	+	+	+	+	+
Ptdss1 variant	c.1058A > G	c.1058A > G	c.1058A > G	c.806C > T	c.794T > C	c.785G > T	C.829T > C	c.794T > C	c.284G > T	c.284G > T	c.284G > T	c.1058A > G	c.806C > T

[1]: (Chrzanowska et al., 1989); [2]: (Saraiva, 2000); [3]: (Wattanasirichaigoon et al., 2004); [4]: (Sousa et al., 2014); [5]: (Tamhankar et al., 2015); [6]: (Whyte et al., 2015); [7]: (Piard et al., 2018); [8]: (Afifi et al., 2019); [9]: (Maden Bedel et al., 2024).

Apart from this, another characteristic of LMS is a severe phalangeal dysplasia affecting mainly the fourth and fifth fingers, with comparatively sparing of the thumb to a certain degree. Our patient presented with several classical features, including global developmental delay, facial malformation, diffusely wrinkled and atrophic skin, cryptorchidism, prominent varicose veins across the scalp and abdomen, cortical thickening of the ribs, and digital abnormalities in the form of middle finger deviation and little finger shortening. These were identical to those found in previously described patients with the same PTDSS1 variant (7). Notably, our patient also exhibited abnormal thyroid function, a characteristic not yet reported, which suggests that PTDSS1 dysfunction might have more widespread systemic effects, possibly even endocrine dysregulation. This observation, while preliminary, points to novel directions of investigation for future research on hormonal implications in LMS.

The PTDSS1 gene is located on chromosome 8q22 and encodes PSS1, an enzyme located in the plasma membrane and endoplasmic reticulum. PSS1 plays a crucial role in the biosynthesis of phosphatidylserine, an essential phospholipid cell membrane component with a basic function in cell signaling, apoptosis, and cell membrane structural integrity (14, 15). Sousa et al. confirmed that GOF variants in PTDSS1 result in elevated phosphatidylserine overproduction and disruption of its negative feedback regulation, thereby inhibiting normal cellular homeostasis (9). This dysregulation is likely accountable for both neurodevelopmental abnormalities, such as intellectual disability, and skeletal pathology, including the progressive hyperostosis of LMS patients.

To date, PTDSS1 gene variants reported to be associated with LMS are c.1058A > G (p.Gln353Arg), c.794T > C (p.Leu265Pro), c.805C > T (p.Pro269Ser), c.785G > T (p.Arg262Leu), c.829T > C (p.Trp277Arg), and c.806C > T (p.Pro269Leu), among others (1, 5, 6–12). All the variants are present in crucial functional areas of the PTDSS1 gene and are expected to result in aberrant enzyme activity and GOF effects. Sugahara et al. demonstrated that PTDSS1 variants can contribute to the osteosclerotic phenotype of LMS by impairing osteoclast formation, multinucleation, and functional activity (16). In addition, Brum et al. characterized CLIC3 as a gene controlling osteoblast differentiation and bone formation through its interaction with NEK9 and PTDSS1. These findings are novel information regarding LMS molecular mechanisms and potential therapeutic targets (17).

As of now, there is no treatment for LMS. Therefore, clinical management remains symptomatic and supportive. For instance, our patient's abnormal thyroid function improved with appropriate medical treatment. However, the hallmark features of LMS like progressive skeletal dysplasia, intellectual disability, and cutis laxa require long-term follow-up and multidisciplinary care. In recent years, gene and stem cell therapies have emerged as promising approaches in development. Gene-editing technologies (like CRISPR-Cas9) have the potential to restore the normal function of PTDSS1 by correcting pathogenic variants. In the same way, stem cell–based therapies, particularly using mesenchymal stem cells with self-renewal and multipotent capabilities, can offer promising strategies for tissue regeneration in LMS. PSS1 selective inhibitors are being developed as prospective targeted treatments, although they remain in the early stages of development (18, 19).

In summary, this report contributes a new case from an underrepresented population, expands the phenotypic spectrum of LMS, and highlights the need to consider LMS in the differential diagnosis of congenital cutis laxa and skeletal dysplasia, particularly in the neonate. Because of the rarity and phenotypic diversity of LMS, this case underscores the importance of genetic testing and global case-sharing in rare disease research. It also offers perceptive guidelines for future investigations in disease mechanisms and therapeutic development.

## 4 Conclusions

In this report, we report the first molecularly confirmed Chinese patient with LMS caused by a heterozygous c.806C > T (p. Pro269Leu) variant in the PTDSS1 gene variant. This report adds valuable information to the several literatures in broadening the phenotypic and genetic spectrum of LMS to the underrepresented East Asian population. Interestingly, our patient presented with a combination of classic and atypical manifestations, including mild intellectual impairment, absence of proximal interphalangeal synostosis, abnormal thyroid function test, and mild cerebral atrophy. These findings expanding knowledge regarding the disease phenotype. These findings highlight the importance of LMS being considered in pediatric patients with classic skeletal and cutaneous manifestations regardless of a deficiency of classic presentations. WES needs to be integrated into the diagnostic process to enable early diagnosis, facilitate proper genetic counseling, and maybe future precision therapies.

## Data Availability

The datasets presented in this study can be found in online repositories. The names of the repository/repositories and accession number(s) can be found in the article/Supplementary Material.
